# Intelligence without neurons: a Turing Test for plants?

**DOI:** 10.1007/s00709-021-01642-0

**Published:** 2021-04-10

**Authors:** Peter Nick

**Affiliations:** Botanical Institute Karlsruhe Institute of Technology, Karlsruhe, Germany

*“*I propose to consider the question, ‘Can machines think?’ This should begin with definitions of the meaning of the terms ‘machine’ and ‘think’. The definitions might be framed so as to reflect so far as possible the normal use of the words, but this attitude is dangerous”. With this proposal, Alan Turing (1950) moves into the question, whether intelligence is bound to neurons. It is remarkable that he begins his seminal work on that, what today is so readily (and inappropriately) called artificial intelligence, with a fundamental definition of terminology. He makes a very important point: the attitude to frame definitions on the “normal use of words”, i.e., to give a new and different meaning to terms that are in everyday use already, is “dangerous”. Indeed. A debate, where terms are ambiguous, will turn ambiguous. This primordial fallacy might contribute a big deal to the controversy about so-called plant consciousness. This debate is overlapping, but not identical to the controversy, whether plants can feel pain, which was thematised in the editorial to the preceding issue (Nick 2021) reflecting on the contributions by Robinson et al. (2021) and Baluška and Yokawa (2021). The question, whether plants can feel pain, and the question, whether plants are endowed with consciousness, seem to be of a similar logical structure. Plants are obviously quite different from us. Can we assume that consciousness, a phenomenon, which is even hard to interpret in ourselves, exists in plants, but in a form that is so different that we cannot easily recognise it as similar? Or does the fact that we cannot recognise this phenomenon in plants without inferring numerous auxiliary assumptions, not simply mean that this phenomenon does not have a counterpart in plants? Again, the old debate about homology versus convergence. To dissect this may be rewarding, irrespective of the outcome, because it brings clarity and advances our understanding of the world and of ourselves. However, the debate has suffered from terminological ambiguity from the very beginning—giving a good example for Turing’s dictum. Keeping this principal problem in mind, it is worth reading two contributions to the current issue addressing the topic of plant consciousness from two opposed viewpoints—Mallatt et al. (2021) take a clear stand against, while Trewavas (2021) is a proponent in favour of plant consciousness.

In their contribution, Mallatt et al. (2021) work systematically through a list of twelve claims. Before doing so, they first try to define their object by drawing a line between something they call “higher consciousness” and “phenomenal” or “primary” consciousness. “Higher consciousness” describes a phenomenon characterised by traits linked with personality, such as the ability to reflect on experiences, recognise the own self in a mirror, or ponder a thought. They concede that also their opponents do not claim that plants are endowed with higher consciousness (which seems to be linked with language). This is not the debate—the debate is about presence of “primary consciousness”, which describes that a living being has “a first-person point of view”. The authors break this relatively vague definition down into two operational criteria. There is a mental representation of the sensed world, and there is affective judgement of situations as “good” or “bad”. They quote then, interestingly, a definition from their opponent, defining primary consciousness “as the capacities to be aware of the environment and to integrate sensory information for purposeful organismal behavior” (Trewavas et al. 2020), and they agree with this definition, specifying that *aware* is transcending the realm of mere mechanistic responses to a stimulus and that *purpose* is transcending evolutionary adaptation in the sense that it is a kind of individual volition. Having defined what they mean by consciousness, they are setting forth to “debunk” that primary consciousness exists in plants. Rather than listing these twelve arguments individually, I will try to group them into logical units:

## Stimulus-response versus consciousness

All living organisms are endowed with the ability to perceive and respond to signals from the environment, and this ability has been central to survival. This fact is not disputed by anybody, but it does not mean that this perception and response is a manifestation of consciousness. The authors work this out using the Cellular Basis of Consciousness Theory (Reber 2016) as example.

## Arguments against a plant neurobiology

Plant cells share with animal cells (as with all other cells) the ability to generate membrane voltage and can under certain circumstances (albeit in very specific cells in very specific plants) even generate action potentials. The existence of a vascular system that not only can transport water and assimilates but also signals has been used to draw an analogy with neurons. However, whether a phenomenon is a signal or a by-phenomenon depends on its “meaning”, i.e., on the question, whether this phenomenon is really used to transmit information. This question remains untouched by the notion that such signals can be emitted from specific sites in the plant (in this context, often the root cap is described as a brain-like structure). It also remains untouched by the question, whether these signals are electrical or chemical. It just should be mentioned in this context that the path of research that led to the discovery of the phytohormone auxin was a similar signal transmission phenomenon from the tip of the irradiated coleoptile to the base of this organ, where growth responds to this signal. Several “debunking” arguments deal with the question to what extent the electric phenomena in plants are homologous with their neural counterpart. The (negative) outcome of this investigation tells that they are not. The phloem and neurons are not even convergent structures. The neurons might just be metaphors for signalling in plants (in a way that is quite similar, “neural networks” are described to describe “learning” in artificial intelligence; see at the end of this editorial).

## Plants can anticipate

The adaptation of plants, not only on an evolutionary scale but also as adapting individuals, is impressive. For instance, root systems can forage limited resources (such as sulphur or phosphorous), and this, at first sight, looks like intelligent, anticipative behaviour. However, as efficient as such responses may be, they are not proof of anticipative behaviour, but can be explained by local responses (increased lateral root formation in response to phosphorous depletion, for instance) and mutual competition of lateral organs for limited resources leading to pattern formation. It was Alan Turing, by the way, who could demonstrate, how simple and local processes (that are indeterminate with respect to the individual outcome, but hard-wired with respect to their mechanism) can generate complex, robust, adaptive, and holistic patterns (Turing 1952). To make this clear, what the authors understand as anticipative behaviour, they describe experiments with spiders and contrast those with experiments on strawberry ramets.

The authors conclude with a delineation between consciousness and learning and point out that learning can be achieved without consciousness. This point is relevant. Operationally, “learning” means that the response to a stimulus depends on stimuli that had been perceived earlier. This is also possible on the base of a stimulus-response machinery, any training of a so-called “neural network” can serve as evidence for this. In the attempt to operationalise “consciousness”, they come up with two criteria. The one is so-called high-capacity operant learning, by which they mean “learning a brand new behaviour that uses one’s whole body”, and the second is image-based consciousness. They locate these (rather tight) criteria in vertebrates, cephalopods, and certain arthropods and find as common theme that all these life forms are highly mobile (although they also state that their criteria *per se* are not bound to mobility). Interestingly, they also conclude that primary consciousness in these three groups of animals has evolved independently, meaning that it is a convergence.

The contribution of Trewavas (2021) can be read as a response, but it also tries to operationalise and to define nomenclature. The author begins with a short remark in favour of plant neurobiology referring to the historical work by Sir Bose on electrical phenomena in plants (Bose 1926), but does not dwell on this point, but instead goes on to discuss the more difficult problem of consciousness searching for a way to treat this independently of the question, whether plants have something like a neural system. He first introduces a terminological clarification, replacing the term “consciousness” by the term of “awareness” (which would correspond to the concept of “primary consciousness” mentioned above). One important argument is that “consciousness” is bound to language, and if you cannot ask somebody, it is not possible to decide from outside, whether “consciousness” is present or not. For this operational reason, he also suggests that the behaviour of non-verbal animals should not be described by the same term used for human consciousness.

As central aspect to define “awareness”, the author sees the ability to assess (he uses the term appraisal) the imprint of the detected external or internal signals. This is a criterion where he is congruent with the definition by Mallatt et al. (2021), where both sides disagree the question, whether such an “appraisal” exists in plants, or whether plants respond merely by a stimulus-response machinery. In support of his case, Trewavas (2021) lists a couple of examples, where the response to a given stimulus depends on absence of presence of other stimuli, which is taken as evidence for associative learning. This statement should be questioned, however. In fact, there are numerous cases, where signalling is cross-connecting. However, at least for some of those listed in this context, for instance, the modulation of phototropism (triggered by blue light) by preceding irradiation with red light, mechanistic, “stimulus-response” explanations have been proposed. Those imply, for instance, changes of receptor abundance and activity (so-called sensory adaptation), or time-dependent limitations in signal transduction (so-called habituation). He makes then an important point: any type of learning is a process with a temporal dimension—temporal contiguity is needed for conditioning. Likewise, associative learning is based on contingency between two stimuli. However, as explained by Mallatt et al. (2021), associative learning does not require consciousness, but can also proceed in a stimulus-response framework, as long as the relationships are adaptable, i.e., prone to temporal change depending on the input. We have spelled this out recently addressing the sensory role of microtubules during cold acclimation (Wang et al. 2020). In a short interlude, the author summarises the arguments to discuss “awareness” and avoid the term “consciousness”. He proposes then the use of Integrated Information Theory (Tononi 2004) as a tool to operationalise “awareness”, mainly with the argument that it is “blind to any requirement for brains, nerves, and synapses” (in other words, he separates the plant neurobiology debate from the plant awareness debate, which is certainly meaningful). As central criterion, he emphasises the existence of information loops, where input is not just generating a stereotypic output, but where signalling is feeding back to input. Although originally coined to describe the recurrent signalling in the human brain, this viewpoint (which is, in fact, a resurrection of cybernetics) can also be used to describe other complex behaviours of adapting systems. The information content Φ_max_ of such a network increases with the density of loops and connections and represents something like a holistic memory of the system. As to transcend the level of loops and interactions, one needs an entity where the history of dynamic interactions of such a system is stored. In humans, we assign this function to the brain. But what about plants, where all the time cells are generated and consumed? There are cells that remain active throughout the life cycle of the plant and give rise to all other cells in the plant bodies—these are the meristems. The author proposes that they are the physical seat of Φ_max_, for instance, by virtue of a contiguous extracellular matrix or by a complex and dynamic system of calcium signalling. He concludes with a metaphor of cars driven either by fossil fuel or by electricity alluding to the different lifestyles of animals (responding by movement) and plants (responding by growth and development, i.e., on a different time scale).

It is always a challenge for editorial neutrality to describe a debate as controversial as this, and it may be helpful to take a little detour, looking at the seemingly different, but related phenomenon of so-called “artificial intelligence”. This technological advance is progressively entering our everyday experience and inspires both awe and fascination, because it is somehow behaving “like us”, but on the other side remains “deeply alien” to us. On the operational level, “artificial intelligence” works with so-called “neural networks”. Ironically, these systems are neither neural nor have they been developed to explain neural systems. McCulloch and Pitts (1943) used the metaphor of neural learning to describe self-learning systems. In principle, such systems consist of three layers of interactors, where input (“stimulus”) and output (“response”) are coupled by a central layer that can interconnect with flexible permeability. This permeability depends on both input and output. Using a training data set as input and a feedback that will minimise the difference between input and output, a system is created that “learns” to reproduce and respond appropriately to a given set of inputs. If the number of elements is increased, the complexity and efficiency of such systems can be impressive and, in some aspect, even overwhelm human capacities. In other words, Φ_max_ can, however only in the trained context, exceed our own Φ_max_. But would we assign to such an “artificial intelligence” any “awareness”? This is to be doubted. Will a description of plant behaviour (or the behaviour of any other life form) by Integrated Information Theory really help us to grasp the essence of “awareness”?

It is a peculiar trait of this debate that the number of review articles vastly exceeds the number of research papers on this matter. To render this debate more fruitful, it should be fed with real-world experiments. To compile experiments conducted in a different context to address different questions will not suffice. Instead, experiments have to be specifically designed for the purpose to verify or to disprove implications derived from such operationalisations as developed in the two contributions by Mallatt et al. (2021) and Trewavas (2021). It may be fruitful to separate the controversy about “plant neurobiology” from the debate about plant intelligence or awareness. It may also be fruitful to develop operationalisations that are on the one hand, appropriate, and on the other hand draw a clear line between a stimulus-response (which can be complex and adaptable) mechanism and “appraisal”. To address his initial questions, whether “machines can think”, Turing arrived at an interesting test, called imitation game. The core of this game is that the experimentator looks at the system, whose “ability to think” is to be assessed, as a black box, and simply asks questions to this system “as if it were” able to think. By skillful questioning, the performance of the black box is compared to that of a true thinker. Rather than recycling the same arguments again and again, it would be worth to develop a non-verbal Turing Test; in other words, we need a Turing Test for plants.
